# Uncovering Immune Niches in Health and Disease Using Spatial Transcriptomics

**DOI:** 10.1002/eji.70185

**Published:** 2026-04-07

**Authors:** Johan Thorsson, Yang Zhao, Eduardo J. Villablanca, Camilla Engblom

**Affiliations:** ^1^ Division of Immunology and Respiratory Medicine, Department of Medicine Solna, Karolinska Institutet and Clinical Immunology and Transfusion Medicine Karolinska University Hospital Stockholm Sweden; ^2^ Center For Molecular Medicine Karolinska University Hospital Solna Sweden; ^3^ Science For Life Laboratory Solna Sweden

**Keywords:** cancer immunology, immune niches, mucosal immunology, spatial transcriptomics

## Abstract

Spatial transcriptomics allows for the investigation of complex cellular ecosystems directly in their native tissues and enables the dissection of immune niches as spatially organized and functionally diverse microenvironments across homeostatic, inflammatory, and malignant settings. In this review, we examine how spatial transcriptomics tools have been applied to interrogate the cellular and molecular architecture of immune niches, including the emerging studies of B and T cell clonal niches. We focus on immune niches in intestinal and tumor tissues due to their importance to both health and pathology, discuss pressing immunological questions these technologies may help to address, and highlight future developments in the field.

## Graphical abstract

Through the lens of spatial transcriptomics and associated tools, novel immune niche constituents can now be resolved. Drawing on gut and tumor contexts, we propose a framework to define immune niches and integrate clonal dynamics, aiming to advance niche‐directed therapies toward precise, context‐specific immune modulation.

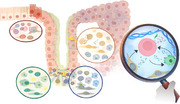

**Abbreviations**
APC: antigen‐presenting cell
APRIL: A proliferation‐inducing ligand
BAFF: B cell‐activating factor
CAF: cancer‐associated fibroblast
CCL: chemokine ligand
CTL: cytotoxic T lymphocyte
CXCL: C‐X‐C motif chemokine ligand
GC: Germinal Center
IBD: inflammatory bowel diseases
IL: interleukin
PC: plasma cell
SPP1: secreted phosphoprotein 1
TAM: tumor‐associated macrophage
TGFß: transforming growth factor beta
Tfh: T follicular helper cell
TLS: tertiary lymphoid structures
Treg: T regulatory cell
TRM: tissue‐resident memory CD8^+^ T cell

## 1 Introduction

The immune system orchestrates complex cellular and molecular interactions to maintain tissue homeostasis and combat disease. These dynamic processes play out across our bodies’ anatomical effector sites, which differ substantially in immune composition and organization, and are heterogeneously organized even within organs, e.g., the proximal‐to‐distal axis of the colon [1] or within malignant tissues [2]. The spatial complexity across tissues underlies the formation of functionally distinct immune microenvironments, which can be defined as immune niches. To understand the diversity and intricacy of immune niches across the body and how they organize to drive protective and pathological immune responses, it is critical to comprehensively capture their molecular underpinnings directly in tissues at scale and high‐resolution.

Spatial transcriptomics is a relatively recent technological breakthrough that, unlike traditional transcriptomic techniques such as bulk or single‐cell RNA sequencing, captures gene expression within intact tissues. In practice, this allows the in situ analysis of molecular and inferred cellular interactions directly in clinical or preclinical tissues, permitting a rich and spatially aware analysis of biological processes directly in their anatomical sites. These capacities have made spatial transcriptomics a transformative tool in immunology, particularly to explore how immune niches form, persist, and function across both homeostatic and pathological settings.

In this review, we discuss the identification of immune niches in health and diseases, suggest a working definition for immune niches, and introduce ways in which researchers use spatial transcriptomics to analyze them. We examine immune niches in intestinal and tumor contexts, focusing on these sites due to the dynamic, clinically relevant, and relatively well‐characterized spatially restricted immune niches that play out in these tissues. We also introduce the emerging field of spatially resolving B and T cell clones within tissues. Finally, we conclude by discussing current limitations of technology and future directions for the field.

### 1.1 What Are Immune Niches?

Despite the frequent use of the term “immune niches” in research, the concept often lacks a precise definition. As described by Lee et al. [3], niches were scientifically introduced in the field of ecology. Here, the term “niche” describes the specific environmental conditions that support a species’ survival and function within an ecosystem. In medical science, the concept of a niche is often used to represent the environment that ensures the function of a specific cell type. The term was first applied to stem cell biology in 1978, when Schofield observed that the bone marrow was more effective than the spleen in supporting hematopoietic stem cells. He proposed that this difference was due to the unique properties of the local microenvironment, thereby coining the “stem cell niche” [4]. The hematopoietic stem cell niche, by far the best‐studied immune niche, provides structural and biochemical cues essential to maintain and regulate stem cells to continuously reconstitute most of the adult immune cells [5]. Since Schofield's time, stem cell niches have been described in various tissues, including the gut [6], skin [7], and central nervous system [8]. Beyond the hematopoietic stem cell niche, immune niches can be more broadly defined as localized environments that influence a range of immune cell functions across their lifespan. These immune niches help shape how our immune cells develop, differentiate, get activated, and ultimately, play out effector functions that both maintain and disrupt our health. Immune niches vary and range from the molecular and cellular cues that either promote fetal‐derived macrophage progenitors to develop into microglia in the brain [9], fuel the expansion of antigen‐specific B cells within germinal centers [10], or sustain long‐lived tissue‐resident T cells in the skin [11].

There are also immune niches unique to the cells of the adaptive immune system, which can be referred to as B and T cell “clonal niches”. B and T cells express clonally unique antigen receptors that confer antigen specificity and conveniently also serve as endogenous cellular barcodes, enabling lineage tracing in space. Throughout a B cell's lifetime, its B cell receptor can also continue to evolve, altering its antigen affinity, and permitting the study of B cell subclones by lineage tracing its receptor sequence [10]. Spatially resolving B‐ and T cell receptor sequences directly in tissues can define how distinct T‐ or B cell clones segregate in specific microenvironments, understand whether certain clones are activated or suppressed, relate clonal distribution with respect to their antigen expression, and identify which receptor sequences are therapeutically or functionally relevant across diseases, including cancer or autoimmunity. Recent work using spatial transcriptomics has begun to explore the immune niches surrounding unique B and T cell clones, mainly in malignant patient samples, but also across lymphoid and mucosal tissues [12, 13, 14]. The concept of “clonal niches” can also be applied beyond B and T cells, such as cancer subclones by mapping copy number variations or mutations [15], or more generally to any cells of the same lineage, defined in situ by exogenously introduced barcoding methods [16].

**FIGURE 1:**
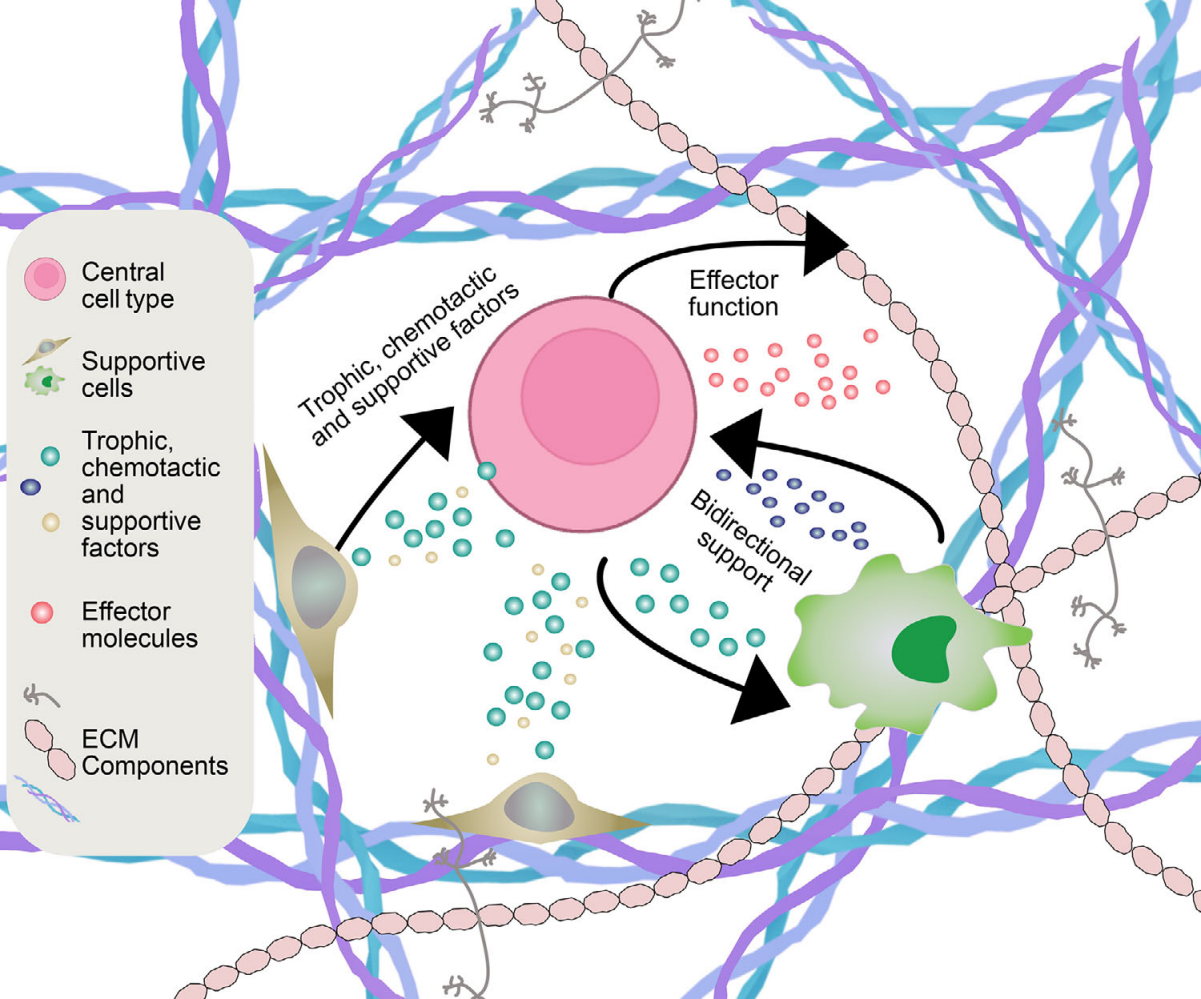
Illustration of an immune niche. A centralized cell type performing an effector function while receiving trophic, chemotactic, and supportive factors from supporter cells. All taking place in an extracellular matrix scaffold.

Here, we aim to refine the immune niche definition beyond “an environment which supports and can influence immune cell function and specialized behaviors” [17]. Building on the macrophage niche definition described by Guilliams et al. [18], we posit that an immune niche should consist of a “central cell” and provide structural support for the cell type it houses, as well as trophic factors for the cell's self‐maintenance, and signals that imprint or sustain cellular function (Figure 1). A central cell could be a long‐lived plasma cell (PC), hematopoietic stem cell, megakaryocyte, resident memory T cell, or tissue‐specific macrophage (e.g., Kupffer cell, osteoclast, or microglia); these cells share that they exert a specialized tissue‐specific function and that they reside long‐term in a well‐defined microenvironment. In addition, we propose that a niche should be reproducibly detected over time at expected sites, emphasising persistence as a defining feature that distinguishes niches from other cellular interactions, such as antigen presentation, which can occur at multiple sites and are typically more transient in comparison [19, 20]. Furthermore, this framework may help to distinguish niches from other concepts, such as cellular hubs, spatial domain, or cellular neighborhoods, which are less clearly linked to a specific function or a centralized cell type as compared with the niche. Other interesting concepts that we considered for a niche definition, while perhaps not yet mature enough to include, are described in Box 1, and can guide how we report and quantify niches in the future. In this review, we focus our discussion on how spatial transcriptomics can be used to investigate immune niches based on our framework.

**Future Niche Definition Concepts**

*Niche Size*

Niches can vary widely in size depending on the species, organ, and type of niche. Knowing the expected dimensions of a given niche would facilitate bioinformatic analyses, help define the anatomical and functional boundaries of niches, and enable comparison across studies, organisms, individuals, and time. However, niche sizes are rarely reported, even though current spatial techniques could easily capture them. One reason may be the lack of clear boundaries for certain niches, since the extent of the supporting environment is often difficult to define. One possible approach is to define niche size based on a central cell type and its physically interacting neighbors within a certain radius. Because physical contact can be transient and detectable only with certain techniques, another option is to consider the niche scaffold size, meaning the spatial domain that forms the home of the central cell population.

*Niche Temporal Stability*

Defining thresholds for the temporal stability of niches is also of interest. We propose that niches should reproducibly be detected at expected anatomical sites, distinguishing them from transient cellular interactions or “aggregates” that are less predictable in location. Furthermore, we believe that stability should be assessed based on the centralized cell type's ability to perform its function within the niche. This definition fits well with tissue resident niches under homeostatic conditions. However, inducible niches that arise during inflammation or malignancy may not fit this framework, as they are usually less stable and less predictable in both timing and location. Therefore, niche longevity or persistence would be important parameters to better quantify, understand, and perturb immune niches and to distinguish these from less structured cellular aggregates, but may be less ideal to formally include in the definition of the niche.

*Bidirectional Support*

Beyond receiving trophic factors from the surrounding scaffold and supporting environment, it has been proposed that centralized cell types can engage in reciprocal communication with their niche supporting environment. For example, in the plasma cell (PC) niche, aged PCs have been suggested to influence myelopoiesis through IL10 and TLR‐dependent mechanisms, illustrating such bidirectional regulation [21]. To include bidirectional support between the centralized cell and its environment could pose a possible new constraint for the immune niche definition. Clarifying the relative interdependence of the central cell and the other constituents is important to functionally define immune niches.

#### 1.1.1 Spatial Transcriptomics

Studying cells within their tissue context is certainly not new, but spatial transcriptomics has substantially expanded our ability to map cellular organization within tissues and characterize cellular niches at high‐resolution and with an increasing ability for unbiased explorations. Conceptually and practically, spatial transcriptomics has advanced the study of immune niches from being able to map the expression of individual or a lower number of genes in tissues, to comprehensively delineating many (or most) genes expressed in defined tissue niches or anatomical structures [22]. There has been consistent technological innovation, leading to the emergence of a wide array of spatial platforms and methodologies [23, 24, 25], the details of which have been comprehensively and expertly reviewed elsewhere [3, 26, 27, 28, 29]. In short, spatial transcriptomics platforms are commonly categorized into two main approaches: sequencing‐based and imaging‐based methods. These strategies are complementary; sequencing‐based methods typically offer broader, unbiased transcriptomic coverage at a lower spatial resolution, while imaging‐based techniques provide higher spatial resolution, albeit with more targeted transcript detection. However, these distinctions have become blurred, as sequencing‐based strategies have approached subcellular resolution and imaging‐based sequencing technologies are rapidly expanding their targeted panels [28]. Sequencing‐based transcriptomics may employ polyA‐ or probe‐based capture of transcripts, the former enabling the discovery of de novo sequences in a species‐agnostic manner. Recent technological advances also enable layering on spatially resolved cell lineage information, such as single nucleotide polymorphism or copy number variations to study in situ tumor cell clonal evolution [12, 15, 30], antigen receptor information for B and T cells to identify and track unique immune cell clones [12, 13, 14, 31, 32], or, in model organisms, introduced cell lineage barcodes to delineate cell development trajectories across tissues [33]. Spatial lineage tracing allows the study of individual cell lineages within their respective tissue niches to better understand cell fate and differentiation trajectories. The growing toolbox of spatial technologies enables researchers to tailor their choice of method based on the biological question at hand, balancing resolution, transcriptome depth, and tissue compatibility to effectively study immune niches across diverse contexts.

### 1.2 Spatial Analysis of Immune Niches

How immune niches are operationally defined and quantified varies across studies and technologies. Niche analysis using spatial transcriptomics typically involves two key steps: first, defining niches based on their spatial location, as well as their molecular and cellular composition; and second, performing bioinformatics analysis to infer biological functions of the identified niches [3]. To explore immune niches systematically using spatial transcriptomics, it is often necessary to apply certain constraints, such as size (Box 1). For data generated by the 10x Visium platform, it is convenient to look at capture spots (∼55 µm in diameter) since it contains several cells, while other platforms with single‐cell resolution enable cell segmentation and thus investigation of finer anatomical regions. Then, single‐cell deconvolution, cell co‐localization, and nonnegative matrix factorization results that are already accessible from standard gene expression analysis can be leveraged for unbiased niche discovery. Single‐cell deconvolution estimates the abundance of cell types within spots, and cell co‐localization quantifies how often different cell types appear near each other. Nonnegative matrix factorization decomposes the gene expression matrix into a small number of gene programs/modules and their spatial weights of each spot, effectively identifying both region‐specific and shared gene programs and making the results biologically interpretable. For instance, Kanemaru et al. profiled cellular niches in the human heart and identified an immune niche, enriched for PCs, within the epicardium–subepicardium structure [34]. These approaches could either help to unbiasedly identify central cells of immune niches, or to describe the cellular composition of an immune niche hosting an already defined central cell, in a more targeted analysis. However, it is also critical to consider aspects beyond cell composition.

Concurrently, rapidly evolving computational tools enable more refined niche identification considering various factors such as cell interactions between neighboring cells and signaling cascades. Methods like NicheCompass frame the niche as the collective impact of neighboring “sender” cells on the gene expression of a central “receiver” cell [35]. In this context, a niche extends beyond just cellular presence to encompass their communication. The COVET (covariance environment) framework offers a more abstract definition, representing a niche by the gene‐gene covariance structure across its constituent cells [36]. This captures the multivariate nature of cellular interactions and defines the niche as a unique “regulatory environment” that shapes a cell's transcriptional state, enabling the discovery of functionally distinct niches. With the emergence of computational, unbiased tools, it is possible to define immune niches at high‐resolution and uncover more biological meanings.

For the identification of B and T cell “clonal niches”, a first step includes the mapping of antigen receptor sequences and grouping of those sequences into clones. Here, based on our proposed niche definition, the individual B or T cell clone would constitute the “central cell”, restricting the definition to cells or shared antigen specificity and ontogeny. (Note, the same “clonal niche” definition could be applied to other cell types if the clonality is captured, e.g., through lineage tracing approaches). Depending on the type of sequencing used (i.e., long‐read versus short‐read), different pipelines can be used to perform basic quality control steps such as demultiplexing and UMI collapsing, followed by clone calling, which in turn can be done with various well‐established methods, including MiXCR [37], IgDiscovery [38], and Immcantation [39]. A key point is that for methods that are not yet at single‐cell resolution, coupling single‐cell spatial clone information with paired single‐cell data or using computational methods to find the most likely receptor pairs can enable follow‐up functional studies of individual receptor sequences [40]. Once the B and T cell clonal sequences have been identified, one can calculate clonal diversity measures across the tissue, identify the clonal gene expression neighborhood by harnessing the paired transcriptome data, and link individual clones with their “clonal niches”. Here, there are numerous computational tools to study antigen receptors defined by single‐cell or bulk sequencing, but fewer ones that have been specifically adapted to spatial analyses.

Following niche discovery, further in silico analysis, such as ligand–receptor investigations and signaling pathway analysis, can characterize the cellular and molecular niche factors that sustain and shape immune niches. For example, Kanemaru et al. [34] revealed that the cardiac PC immune niche was also enriched for the chemokine ligand 28 (*CCL28*), B cell activating factor (BAFF, *TNFSF13B*), and tumor necrosis factor ligand superfamily member 13 (*TNFSF13*, also known as A proliferation‐inducing ligand, APRIL), which recruit and maintain PCs. Similarly, Kuppe et al. [41] revealed the interactions between Secreted phosphoprotein 1 (*SPP1* ^+^) macrophages and myofibroblasts within the myeloid–fibroblast niche in human myocardial infarction across disease stages with upregulated pathways of *SPP1*, Platelet‐derived growth factor D (*PDGFD*), and transforming growth factor beta (*TGFB1*), offering insights into the pathogenic mechanism of the disease.

**FIGURE 2:**
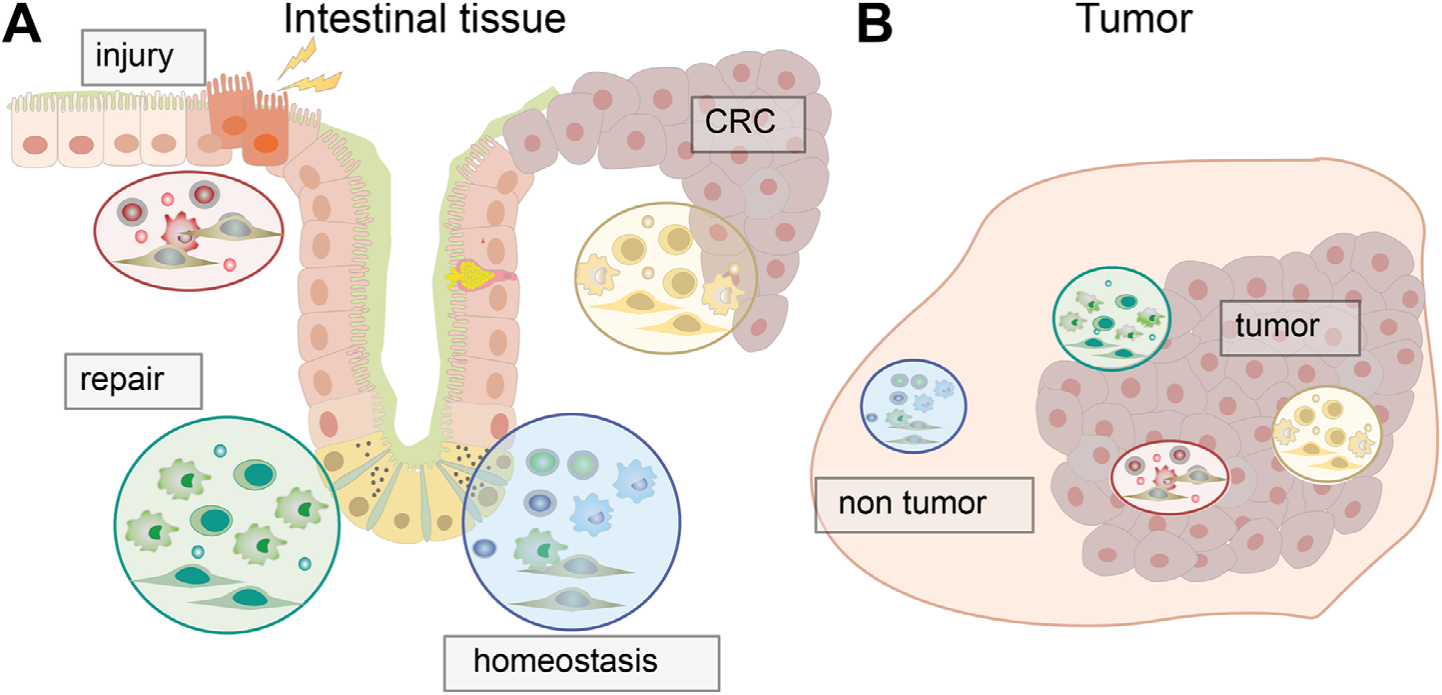
(A) Schematic representation of immune niche distribution across the intestinal mucosa. (B) Schematic description of immune niche organization within the tumor microenvironment. In A and B, different niches are highlighted in colored circles. CRC: colorectal cancer.

Taken together, spatial transcriptomics and associated bioinformatics tools enable a high‐resolution map of immune niche constituents (including the central cell), antigen receptor information when applicable, and transcriptional activities directly within the tissue. Advanced computational tools, such as NicheCompass and COVET, enhance niche characterization by integrating spatial, molecular, and signaling data, revealing the biological roles and potential regulatory mechanisms of immune niches. In the following sections, we will describe multiple examples where spatial transcriptomics elucidated the composition of distinct and diverse immune niches. We will focus on the gastrointestinal tract as an example of highly adaptable niches that are continuously challenged by the environment and tumors, as an example of ectopic niches associated with disease (Figure 2A,B).

### 1.3 Immune Niches in the Gastrointestinal Tract and Cancer

Spatial transcriptomics has been successfully applied to immune niches in various tissues, with many studies focusing on the gastrointestinal tract and the tumor microenvironment. These tissues represent two complex and dynamic environments in which specialized immune niches serve various and distinct but also shared roles. For this reason, we considered them useful reference points for highlighting how spatial transcriptomics can be used to investigate niche‐specific biology. In the gut, the gut‐associated lymphoid tissue forms the largest immune organ and serves as the first line of defense against pathogens and harmful agents [42]. Maintaining barrier homeostasis depends on distinct immune niches shaped by their local microenvironment, creating a heterogeneous tissue landscape visible even through classical histopathology, such as Peyer's patches, granuloma formation, or lymphocytic infiltration in tumors [43, 44, 45]. Similarly, the tumor microenvironment consists of diverse populations of cancer, stromal, and immune cells whose spatial organization and interactions influence tumor progression, therapeutic response, and patient outcome [46, 47, 48]. Thus, we can contrast stable niches involved in homeostasis with niches associated with pathogenesis, which may be less stable due to their inducible nature. For our discussion, we both consider tumors that occur within and outside the gastrointestinal tract, and to limit the scope, we focused on spatial transcriptomics studies that investigated the more well‐characterized macrophage, T cell, and B cell niches.

#### 1.3.1 Macrophages

Macrophages are key tissue‐resident innate immune cells well‐known to influence and be influenced by their local microenvironment. Across the body, tissue‐resident macrophages perform diverse and vital functions, such as bacterial clearance, bone remodeling, and regulation of the nervous system. Depending on their functions, macrophages occupy distinct tissue niches. As noted above, the definition of tissue‐resident macrophage niches is already well‐established and served as a springboard for this review [18]. However, due to their complexity, macrophage subsets are not easily captured by single or a low number of markers typically used for immunofluorescence or flow cytometry; thus, it has been technologically challenging to map macrophage heterogeneity directly in tissues. Spatial transcriptomics, due to its capacity to simultaneously capture cell types alongside their transcriptional cell state, permits identifying complex macrophage states within their niches. Such technology that quantifies transcriptional activity in situ is also helpful to capture macrophages’ dynamic responses to their local environment and infer the signals that macrophages receive from and transmit to their cellular neighbors.

Garrido‐Trigo et al.’s [49] study employed these features of spatial transcriptomics by investigating multiple gastrointestinal macrophage niches in Inflammatory Bowel Disease (IBD) patients using both single‐cell transcriptomics and image‐based transcriptomics with a 1000‐gene CosMx panel, enabling label transfer from single‐cell RNA sequencing to spatial coordinates (Box 2). Macrophage subsets were then mapped to either healthy or inflamed regions of the colon at single‐cell resolution using deep transcriptional profiles, providing a detailed map of the macrophages’ intestinal landscape. In healthy colon tissue, two main resident macrophage subsets were defined in detail and spatially localized in the lamina propria below the apical epithelium. Their respective niches, including local function and potential supporting interactions, were less explored in this study. In IBD patient tissue, inflammation‐associated macrophage subsets showed marked transcriptional heterogeneity across individuals, consistent with variation in their origins and local tissue context. Notably, the authors mapped a macrophage subset, referred to as “inflammation‐dependent alternative” (IDA) macrophages, that was frequently co‐localized with inflammatory fibroblasts, which in turn expressed colony‐stimulating factor (CSF) 2 and 3 and prostaglandin‐pathway genes, indicating potential fibroblast‐to‐macrophage activation signals. IDA macrophages appeared either within granulomas or directly beneath the epithelium. The latter sub‐epithelial IDA macrophage population expressed high levels of EGFR‐ligands, which is important for epithelial barrier repair, implying a macrophage‐to‐epithelium signaling axis that may support epithelial regeneration [50]. This points to a potentially inducible regenerative niche that emerges during barrier disruption, which can occur during IBD, although further studies are needed to elucidate dependencies of this macrophage subset, their niche formation, and stability during inflammation. Combined, in this study, spatial transcriptomics enabled spatially resolving diverse macrophage cell states across the intestine, identified a new macrophage subset with an inferred role in epithelial regeneration, and uncovered putative macrophage‐fibroblast cross‐talk. In the framework of our immune niche definition, the macrophage, as a central cell type, occupies several different niches in the gut, with distinct cell neighbors, molecular environments, and inferred functions.

Like intestinal macrophages, tumor‐associated macrophages (TAMs) exhibit diverse cell states [51, 52] and a range of functions, including promoting tumor progression, by regulating angiogenesis and metastasis, and contributing to antitumor immunity by priming and restimulating T cells and phagocytosing cancer cells [53]. Combined, TAMs are regarded as central orchestrators of tumor initiation, progression, metastasis, and treatment response. Using spatial transcriptomics (Visium and Visium HD), *SELENOP*‐expressing macrophages were mapped in lung and colorectal cancer [54]. This subset has been associated with responsiveness to neoadjuvant chemoimmunotherapy and was either co‐localized with Cancer‐associated fibroblasts (CAFs) or situated within tertiary lymphoid structures (TLSs) [55]. Furthermore, TAMs can be recruited and receive support from trophic factors provided by CAFs, such as *CSF1* and *CXCL12* [56]. Additionally, Oliveira et al. [54], using Visium HD and Xenium, identified *SPP1*+ macrophages around the tumor periphery in colorectal cancer patient samples. Using ligand–receptor interaction inference analysis, they showed that *SPP1*+ macrophages interacted with surrounding T cells and tumor cells through an SPP1–CD44 axis. *SPP1*+ macrophages also co‐localized with CAFs, which in turn highly expressed *MMP11* that can break down the extracellular matrix, relating to worse prognosis and tumor promotion. CAFs can produce cytokines and chemokines to polarize macrophages toward a more immunosuppressive type [56]. Conversely, macrophage‐derived signals can also promote immunosuppressive CAFs [57]. Both TAMs and CAFs could, in theory, be regarded as a central cell type in a tumor‐promoting niche, since they appear to contribute to each other's niche, providing bidirectional support to each other. Macrophages can take on both pro‐tumor and antitumoral roles; a study by the Pittet lab found that *CXCL9* versus *SPP1* expression separated two distinct macrophage subtypes, with a *CXCL9^−^* versus *SPP1‐*enriched molecular signature relating to antitumor immunity versus tumor promotion, respectively. Using RNA in situ hybridization and CosMx, the authors found that these subsets spatially localized with macrophages of similar phenotypes, respectively, and appeared in distinct cellular and molecular neighborhoods related to tumor control (*CXCL9* ^+^ macrophages) versus tumor progression (*SPP1* ^+^ macrophages) [58]. The same study also found that the *CXCL9:SPP1* TAM polarity was broadly useful as a prognostic indicator for cancer patient disease outcome across cancer types, suggesting high relevance of these signaling pathways for tumor control. In these studies, spatial transcriptomics enabled capturing TAM niches across tumor types and inferring cell‐to‐cell interactions with their niche neighbors.

Macrophage niches are thus shaped by distinct contextual demands in the gastrointestinal tract versus tumors, spanning homeostasis and inflammation, but they also share similarities across the tissues. For instance, macrophages and fibroblasts frequently co‐localize in the gut as well as across different cancer types, indicating a stable cellular interdependency [18, 50, 55]. Spatial investigations in healthy gastrointestinal tissue revealed a relatively predictable organization of macrophage subtypes, whereas inflammatory settings were marked by a more heterogeneous pool of macrophages with divergent transcriptional profiles. Notably, one inflammation‐associated macrophage niche appears to support epithelial regeneration and may be sustained by fibroblasts, like in tumors. These findings exemplify regenerative macrophage–fibroblast niches that can be beneficial during tissue repair but also co‐opted to support tumor development. While these niches are reproducibly detected and thereby indicative of stable arrangements in the tumor context, more functional testing would be needed to evaluate the stability of the regenerative niche in the IBD context and in the context of tumor formation. Macrophage niches in cancer and IBD patients are also highly diverse across patient groups, and further spatial transcriptomics studies may help to stratify disease outcomes based on differences in macrophage transcriptional profiles and spatial arrangements.

#### 1.3.2 T Cells

T cells comprise a diverse lineage with distinct phenotypes and functions across tissues, including CD4^+^ and CD8^+^ subsets. CD4^+^ T cells differentiate into specialized helper and regulatory subsets, such as T helper (Th) 1, Th2, Th17, T follicular helper cells (Tfh), and T regulatory cells (Tregs) [59]. They coordinate the cytokine milieus, provide help to B cells and CD8^+^ T cells, and enforce or relax immune tolerance. CD8^+^ T cells, in turn, act as key cytotoxic effectors and can directly kill infected or malignant cells [60, 61]. T cells are mobile cells that circulate throughout the body during their development, activation, selection, and differentiation, ultimately performing critical effector functions in tissues. Capturing their phenotypes and niche interactions in situ in an unbiased way is therefore important, but can be challenging, due to their smaller size and somewhat sparser transcriptional activity on a per‐cell basis compared with other cells, for example, macrophages. Likewise, investigating the spatial distribution of ontogenically related T cells, which thus share the same antigen specificity and antigen receptor sequence, is important but has been technically difficult before the development of spatial T cell receptor tracing tools. Below, we will highlight a selection of studies that have harnessed spatial transcriptomics to study T cell niches.

One of the best‐characterized examples of an immune niche involved in maintaining gut immune balance involves Tregs, which are effective in controlling gut homeostasis [62]. Gu et al. [63] investigated mechanisms underlying immune tolerance by characterizing what the authors referred to as “the effector regulatory T cell (eTreg) microniche”. This study used two‐photon‐photo‐activation labeling followed by single‐cell RNA sequencing (NICHE‐seq) through integration with Visium [64], which enabled better resolution of the transcriptional landscape of the lamina propria. The authors then mapped cell types into regions in the lamina propria defined by manual annotation and NMF clustering, comparing anatomical regions using transfer of antigen‐specific CD4^+^ T cells reactive to *Helicobacter hepaticus* in settings of tolerance and inflammation. Focusing on eTregs as the central cell, the authors identified subsets of antigen‐presenting cells (APCs) providing support to eTregs in the lamina propria through various chemokine–chemokine receptor and integrin pairs in the tolerogenic model, which were confirmed by in vivo live imaging. This niche promoted eT_reg_ stability, proliferation, and displayed a distinctive effector gene signature of amphiregulin, granzyme B, and interleukin (IL)‐10, which are important genes connected to enhanced suppressor functions. Furthermore, inflammatory conditions disrupted this immunoregulatory environment through the infiltration of various cell types, such as CD103e^+^ SIRPα^+^ dendritic cells, which contributed to the breakdown of the tolerogenic niche. Here, the authors benefited from the possibility to survey broader tissue regions using spatial transcriptomics and in a more unbiased way capture cell–cell ligand–receptor interactions.

Tregs are also well‐established orchestrators of immunosuppression within the tumor microenvironment. Song et al. used seq‐based spatial and single‐cell transcriptomics, flowcytometry, multiplex immunohistochemistry, and functional in vitro studies to identify a more immunosuppressive terminal eTreg expressing IL‐1β [65]. In this case, the sequencing‐based spatial transcriptomics alone was not used to capture the niche interactions with the particular terminal eTreg subset as the central cell, likely due to the lower sensitivity in gene capture with these platforms and the nuanced transcriptional differences between these subsets. Instead, the authors used a combination of multiplex immunohistochemistry and single‐cell transcriptomics, the latter of which captures higher transcript count per cell compared with spatial transcriptomics, and found that terminal eTregs co‐localized with TAMs and inferred multiple ligand–receptor interactions, potentially sharing similarities with the eTreg niche described in the gut. Taken together, this study illustrates the need to use multiple strategies to harness technological strengths (e.g, sensitivity to capture cell states in single‐cell transcriptomic datasets and location across cell types in spatial transcriptomics), while overcoming inherent technical limitations.

Whether Tregs establish stable, reproducible immune niches in cancer remains to be fully addressed. Immune checkpoint blockade can dampen Treg suppression for many tumor types, which can benefit antitumor immunity; however, limiting Treg functions systemically may also contribute to treatment‐induced immune‐related adverse effects, such as colitis [66]. Better understanding of Tregs and their niches across tissues is needed to explore whether therapeutically targeting Treg niches locally in the tumor, for example, by intratumoral delivery of immune checkpoint blockade, would enhance antitumor immunity while limiting debilitating side effects. Future spatial transcriptomic studies integrating high‐resolution, longitudinal sampling, and functional perturbation will be essential to determine whether Treg‐enriched regions represent true immune niches with stable architecture and defined cellular dependencies, or if they reflect more dynamic, transient accumulations of suppressive cells.

Cytotoxic T lymphocytes (CTLs) play important roles in barrier maintenance and also in antitumor immunity. For example, tissue‐resident memory CD8^+^ T cells (TRMs) help protect the epithelial barrier and have been extensively studied for their role as surveyors and resident effector cells in tissues since their discovery in the late 2000s to early 2010s [67, 68]. In the small intestine, single‐cell studies of CD8^+^ TRMs identified two distinct subtypes, which differed in effector and memory potential [69]. Using both high‐resolution sequencing‐based transcriptomics and image‐based spatial transcriptomics, Reina‐Campos et al. [70] located distinct anatomical niches for these subtypes along the crypt–villus axis that imprint unique transcriptional and functional programs onto TRMs. The upper and lower villus environments created a division of labor, with effector‐like TRMs positioned for immediate defense at the tip neighbored by enterocytes in an IL‐7 and IL‐15‐rich environment. In contrast, progenitor‐like TRMs that provide long‐term memory are found at the base in an area enriched for fibroblasts, B cells, and CD4^+^ T cells and chemokines such as CXC chemokine ligand (*Cxcl9)* and *Cxcl10*. Importantly, gene perturbation studies indicated that TGFß signaling promoted CD8^+^ TRM differentiation toward an effector‐like state. Taken together, the authors discovered a previously unknown zonation of two CD8^+^ TRM subtypes, linking tissue location to the imprinting of cellular function. Here, their discovery was enabled by “gating” on different tissue sites and capturing their respective niche transcriptional states; a task that would have been challenging with other types of technologies.

Tumor‐infiltrating T cells, particularly CTLs, mediate antitumor immunity, are the main targets of current immunotherapies, and have been the subject of intense investigation in the field. Many studies using single‐cell and spatial transcriptomics have documented that CTLs have distinct cell states and spatial infiltration patterns [71, 72]. Their presence and spatial organization within tumor tissue also often correlate with favorable patient outcomes [72]. Tumor‐infiltrating T cells frequently aggregate in distinct niches at tumor margins and within the tumor itself, where local activation, clonal expansion, and crosstalk with other immune or stromal cells occur. Pelka et al. [73] leveraged the GeoMX platform to show that in human colorectal cancer, a positive feedback loop involving *IFNγ* and chemokines, *CXCL9/10/13*, sustained T cell recruitment and activation in spatially defined immune niches sharing chemotactic signals with the gastrointestinal crypt CD8^+^ TRM niche. Using Slide‐seq and spatially resolved TCR analysis in melanoma, Liu et al. [12] revealed that single T cell clones reside in specific microenvironments, shaped by distinct migration and ferroptosis signatures in nearby myeloid or tumor cells. In this study, the authors developed a spatial transcriptomics‐based tool to couple transcriptional information with de novo captured T cell lineage information to infer clonal niches exhibiting distinct pathway activation, the latter of which may not have been easily captured by more targeted methods.

These studies demonstrate a wide variety of spatial investigations into distinct T cell niches using multiple spatial transcriptomics methodologies. T cell niches in the gut and tumor have similarities, such as shared chemotactic signals for CD8^+^ T cells or overlapping spatial neighbors in Treg niches. Understanding differences and similarities between these niches could help facilitate differential targeting. For instance, we know that tumor Treg niches could be distinguished by supporting cell types, such as TAMs, or specific signals like hypoxia, metabolic stress, and tumor‐derived cytokines, which appear to be absent in homeostatic intestinal tissues [65]. Targeting these molecular and spatial signatures could enable the design of therapies that selectively disrupt tumor immunosuppression while preserving intestinal tolerance, thereby reducing immune‐related adverse effects such as checkpoint blockade‐induced colitis.

However, the inferred T cell niche interactions still require validation (e.g., through in vivo imaging in [63]), showing the importance of combining spatial transcriptomics approaches with other methodologies. Applying higher‐resolution platforms such as Visium HD, Xenium, Slide‐seq, or Stereo‐seq for single‐cell annotation can more precisely define local microenvironments of distinct T cell subsets, as illustrated by Reina‐Campos et al.’s [70] mapping of two distinct CD8^+^ TRM populations along the crypt‐villus axis. Combined, most of the T cell niches we have described here in the gut context are considered relatively stable, as they are detected under homeostatic conditions and are disrupted primarily upon inflammatory or experimental perturbation. In contrast, T cell niches in tumors are typically identified in specific patient subgroups and disease contexts; while they can be reproducibly detected, their long‐term stability remains unclear and will require systematic perturbation‐based and longitudinal analyses. Nevertheless, several niche‐defining features are shared across these contexts, including the co‐localization of Tregs with APC and the use of common recruitment and positioning cues for cytotoxic T cell niches, such as CXCL10‐driven chemotaxis.

#### 1.3.3 B Cells

B lineage cells, comprising both B cells and PCs, are the main players of humoral immunity, but have traditionally been understudied in tumor immunology and mucosal immunology compared with T cells [74, 75, 76]. Studies of tertiary lymphoid structures (TLS) have rapidly expanded the interest and understanding of B cells, particularly in cancer immunology. Broadly, B cell activation, clonal selection, and differentiation into memory B cells and PCs are tightly orchestrated in spatially restricted zones. Therefore, it is critical to study the evolution of B cell lineage cells within their native tissue context and in relation to their cellular and molecular surroundings. Spatial transcriptomics lends itself to capturing complex B cell states, particularly in lymphoid tissues, and unique B cell receptor sequences to enable lineage tracing [13, 77]. Furthermore, PCs, terminally differentiated antibody‐secreting cells of the B cell lineage, are particularly easy to identify using spatial transcriptomics due to high transcriptional activity during antibody production. This feature facilitates both identifying where antibodies are being produced and their respective isotype, tasks that would be challenging using spatial proteomics, requiring multiple markers not typically included in standard immunofluorescence panels. For instance, antibodies targeting the protein CD138 are often used to stain for PCs in tissue sections; while CD138 is a useful marker in lymphoid tissues, it is also highly expressed by epithelial cells, including many cancer cells, making it less suitable to study PCs in tumors and the intestine.

In mucosal tissues, B lineage cells provide antibody‐mediated protection against microbes and form important immune niches with their molecular and cellular neighbors [76]. In the intestine, PCs secrete antibodies against microbes to maintain homeostasis, and “long‐lived PC niches” support the secretion of IgA antibodies into the intestinal lumen and the long‐term survival of PCs [78]. Remarkably, PCs can persist for decades in human intestines [79]. IgG‐secreting PCs identified in patients with IBD correlate with increased disease severity, and PC numbers vary among IBD patients, suggesting their involvement in pathology [80, 81]. The molecular organization of the PC survival niche resembles that of its bone marrow counterpart, which has been extensively explored [82]. As in the bone marrow, stromal cells, e.g., intestinal epithelial cells in the gut, support PC survival by producing key factors such as BAFF and APRIL [78]. The PC niche in tumors is less well characterized, with regard to its molecular and cellular constituents, generalizability across tumors, stability, and potential longevity.

Spatial transcriptomic mapping of the naïve murine colon has resolved regions associated with PCs [1]. In a recent preprint, Yang et al. investigated the long‐lived PCs’ niche in the mouse intestine using high‐resolution image‐based spatial transcriptomics (MERFISH) [32]. The biggest proportion of PCs was localized to the middle portion of the villus, associated with a high CCL25 gradient. Importantly, this study extended the MERFISH method to investigate PC clonal diversity within their tissue microenvironments in the ileum. Their “B cell receptor ‐MERFISH” approach found that unique combinations of heavy and light chain V gene usage (approximating individual B cell clones sharing the same ancestry) were distributed across the proximal to distal regions of the ileum. Although the authors’ approach does not capture the full B cell receptor sequence to enable de novo CDR3 capture or mapping of somatic hypermutation, it offers a compelling, high‐resolution manner to link PC clonality and lineage to their local niche. In colitis models, infiltrating B cells are captured using platforms such as Visium [83], but B cells within TLS or B cell follicles can be more difficult to detect, as cell permeabilization and cell segmentation are more challenging in densely packed cellular regions. Combined, spatial transcriptomics may help uncover how PC niches are dynamically changed between health and diseases, such as IBD or gastrointestinal cancer, and provide information on potentially pathogenic PC clones, including their antigen specificities, which could offer new vantage points for therapies.

In cancer, B lineage cells can perform a range of functions, including antigen presentation to T cells, antibody production, and coordination of the tumor microenvironment [84]. Generally, B cells cluster within tumor tissues as aggregates or highly organized TLSs fueling B cell maturation, differentiation, and T cell‐mediated immunity [83], whereas PCs, terminally differentiated antibody‐secreting cells of the B lineage, are more frequently located at the periphery of TLSs within the tumor stroma or interspersed among and outside tumor cell aggregates [85]. The presence of TLSs in cancer has been associated with improved prognosis and enhanced responsiveness to immune checkpoint blockade across a wide range of cancer types [86]. TLSs are also commonly observed in IBD, but they are associated with more severe forms of disease [76, 87, 88]. By our immune niche definition, we would consider TLS as a platform that houses several interconnected immune niches, each with its own central cell, structural support, and functionality, including germinal center (GC) B cell niches. GC B cells get structural and trophic support from follicular dendritic cells, which retain the antigens essential for B cell selection, and Tfh, which deliver co‐stimulatory signals and cytokines to promote B cell survival and differentiation [10]. GC B cell niches are reproducibly detected within what are often called “mature TLS” in tumors, although it is challenging to pinpoint “maturity” in patient samples as they often lack temporal dynamics [83]. Using spatial transcriptomics, Li et al. found supporting factors *CXCL13*‐*CXCR5*, follicular dendritic cells, and structural support of vasculatures within GC B cell niches in tumor‐associated TLSs [89]. Finally, we developed and applied spatial transcriptomics‐enabled lineage tracing of B lineage cells to breast tumor tissue, which highlighted the potential to capture distinct B cell clones adjacent to different tumor regions [40]. Further studies will help to clarify B‐cell clonal dynamics and niches across TLS and the tumor microenvironment.

The pathological role of TLS in IBD has not yet been extensively explored using spatial transcriptomics, but could help to understand TLS‐associated B cell niches’ contribution to pathology and whether these niches differ from their counterparts in the tumor setting. B cells can also infiltrate and form aggregates in the inflamed intestine, independent of TLS [90, 91]. Frede et al. [92] used sequence‐based spatial transcriptomics combined with single‐cell RNA‐sequencing to explore the impact of B cell expansion during inflammation on epithelial regeneration. B cells physically disrupted the regenerative crosstalk between stromal compartments and the intestinal stem cell niche, thereby impairing mucosal healing. For this study, spatial transcriptomics specifically helped to identify and focus follow‐up studies on B cells as players in the stroma‐epithelial cross‐talk based on their tissue location. Altogether, exploring B cell niches and TLS in particular, both in cancer and IBD, may represent alternative therapeutic strategies, but further studies are needed.

#### 1.3.4 Summary

In this section, we highlighted six main features of mapping immune niches in the gut and tumor tissues using spatial transcriptomics. First, spatial transcriptomics permits taking an unbiased approach toward broadly defining a particular area of interest by “gating” on tissue sites and defining the transcriptional, cellular, and inferred functional states. This was most obviously shown by identifying distinct T cell zones in the intestinal crypt and villi [70]. Second, it allows to simultaneously map many complex cell states not easily defined by single markers, which was exemplified by several macrophage niche studies. Third, due to broad coverage, it can capture inferred cell‐to‐cell communication by identifying neighboring cells to the “central cell” in the niche, alongside its ligand–receptor pairing, as illustrated by the discoveries of macrophage‐fibroblast interactions in tumors and gut tissues, as well as T cell‐chemokine signals. Fourth, spatial transcriptomics helps to prioritize which cell types to investigate in functional studies based on their location within a given niche, as shown by Frede et al. [92], which may limit “noise” in datasets when many cell types change simultaneously. Fifth, the coupling with de novo captured sequences permits mapping clonal lineages of B and T cells in human tissues within their anatomical niche, which was shown by mapping T cells and PCs in tumors and gut [40, 93, 94], respectively. Finally, spatial transcriptomics helps to uncover specific pathway activation hubs, which may not be restricted to individual cell types, but could simultaneously involve multiple cell types in distinct sites, such as in specific tumor locations [95] or at the base of the intestinal crypt [70].

**BOX 2 | Niche components inferred by spatial transcriptomics in selected studies**

**Table:** 

Niche components/Study	Regenerative IDA macrophage niche, Garrido‐trigo et al. [49]	Tumor‐associated macrophage niche, Oliveira et al. [54]	CD8 TRM niches, Reina‐Campos et al. [70]	Clonal plasma cell niche, Yang et al. [32]
Key findings	A putative macrophage niche involved in epithelial regeneration for patients with IBD	*SELENOP* ^+^ and *SPP1* ^+^ macrophage niches with M2‐like tumor‐promoting phenotype	Zonation of CD8^+^ TRM subsets along the crypt to villus axis	Approximation of clonal plasma cell mapping, abundance linked to microbiota exposure
Central cell type	Inflammation‐dependent alternative (IDA) macrophages	*SELENOP* ^+^ and *SPP1* ^+^ macrophage	Both effector‐like CD8^+^ TRM: high expression of *Gzma, Gzmb*, and *Itgae*, and progenitor‐like CD8^+^ TRM: *Tcf7* and S*lam7*	Plasma cells
Location	Colon stromal compartment and apical epithelium	Tumor periphery	Upper and lower villi of the small intestine	Ileum, with the highest proportion in the middle of the villus
Inferred function	Regenerative	Tumor‐promoting	Effector like CD8^+^ TRM: Cytotoxic potential progenitor like CD8^+^ TRM: Memory potential	Humoral immunity
Niche cell composition (excluding central cell)	Inflammatory fibroblasts	Cancer‐associated fibroblasts, tumor cells, T cells	Effector like CD8^+^ TRM: Enterocyte Progenitor like CD8^+^ TRM: fibroblasts, B cells, and CD4^+^ T cells	Not addressed
Niche‐associated molecular signals	Myeloid colony‐stimulating factors (*CSF2* and *CSF3*) and prostaglandin‐pathway genes expressed by surrounding fibroblasts	Tumor‐ and stromal‐derived signals, for example, *TGFBI, REG1A*, sustain macrophage state	Effector like CD8^+^ TRM: microenvironment was enriched in IL‐7 and IL‐15. Progenitor like CD8^+^ TRM: This niche is enriched in Chemoattractant signals, including *CXCL9* and *CXCL10*, thereby recruiting *CXCR3^+^* T cells during early inflammation.	The chemokine CCL25 gradient correlated with higher plasma cell abundance in the middle villus.
Bidirectional support	Not assessed	Not assessed	Not assessed	Not assessed
Niche size	Not assessed	Not assessed	Not assessed	Not assessed
Temporal dynamics	Not assessed	Not assessed	In a mouse model of LCMV infection with adoptively transferred P14 CD8^+^ T cells assessed at multiple time points, this zonation was evident by 90 days post infection, but not assessed for stability at later time points.	Not assessed
Feature of spatial transcriptomics analysis that enabled this study	Spatial transcriptomics profiling helped to map transcriptionally complex macrophage subsets (not readily identified by single or a low number of markers). In this context, label transfer from single‐cell transcriptomic datasets onto single‐cell‐resolution spatial transcriptomic data provided a powerful approach for studying macrophage transcriptional states and niche organization. Spatial transcriptomics analysis also enabled inferring putative signaling molecules between distinct macrophage subsets and fibroblasts or epithelial cells, generating further hypotheses on cell–cell communication within the niche.	Spatial transcriptomics enabled accurate identification and mapping of transcriptionally diverse macrophage subsets within their native tissue architecture. Furthermore, preserving this spatial context enabled detailed pathway analysis to investigate cellular crosstalk within the microenvironment, specifically highlighting complex interactions and communication networks between localized macrophages, T cells, and tumor cells.	CD8^+^ TRMs zonation along the crypt‐villus axis could, in principle, be detected by spatial proteomics using known memory or effector markers. Yet, spatial transcriptomics enabled simultaneous investigation of the underlying interactions and signaling programs driving differentiation, whereas other methods, such as laser microdissection, can also reach deep transcriptomic or proteomic profiles, to sample many regions is arduous and lower throughput https://pmc.ncbi.nlm.nih.gov/articles/PMC13001730.	Plasma cells capture using transcriptomics methods is facilitated by their high transcriptional activity, which readily also reveals isotype information. Here, lineage tracing in situ was made possible using the in‐house method BCR‐Merfish, which captured clonal information. Comparatively, plasma cell detection and environmental profiling using spatial proteomics approaches would require multiple markers, and lineage tracing would not be feasible.

## 2 Current Challenges and Future Perspectives

Spatial transcriptomics, with its broad coverage across relatively large tissue regions, is a powerful exploratory tool offering investigation of features not yet attainable with other modalities. Despite the potential of spatial transcriptomics to delineate immune niches within tissues, there are still several technical challenges and limitations that need to be considered. First, spatial techniques vary in their sensitivity, resolution, and tissue coverage. However, both sequencing‐ and imaging‐based platforms are increasingly converging toward single‐cell spatial resolution, higher sensitivity, and extended transcript/tissue coverage [54, 96], such as for Visium HD (both probe‐based and 3’ ployA capture‐based), Slide‐tags [97], and DBiT‐seq [98]. Second, most current spatial transcriptomics methods provide only static, two‐dimensional snapshots of a tissue, which limits our ability to capture spatiotemporal and three‐dimensional dynamics of immune niches. Computational tools like stLearn [99], SPATA2 [100], Cell2fate [101], and SIRV [102], built to infer temporal dynamics through spatial trajectory analysis or RNA velocity, help approximate the “temporal” dimension. Similarly, both experimental and computational tools seek to reconstruct the three‐dimensionality of tissues by, e.g., by consecutive sectioning of tissues [103] or through analytical tools such as MATRICS‐A [104] and X‐Pression [105]. Third, mechanistic studies remain critical to address the functional contribution of each component to the immune niche and its role in maintaining health or driving pathology. Given the increased number of features identified within niches, accelerating the throughput for perturbation studies is critical. Although not available for every tissue type and disease setting, organoid or multicellular system platforms employing gene‐editing or drug screening approaches could help test at least some function of individual components in systems of reduced complexity [106]. Combined, these analytical and experimental advancements can help to overcome some of the current technical limitations of spatial transcriptomics and to better capture immune niche dynamics and functionalities.

**FIGURE 3:**
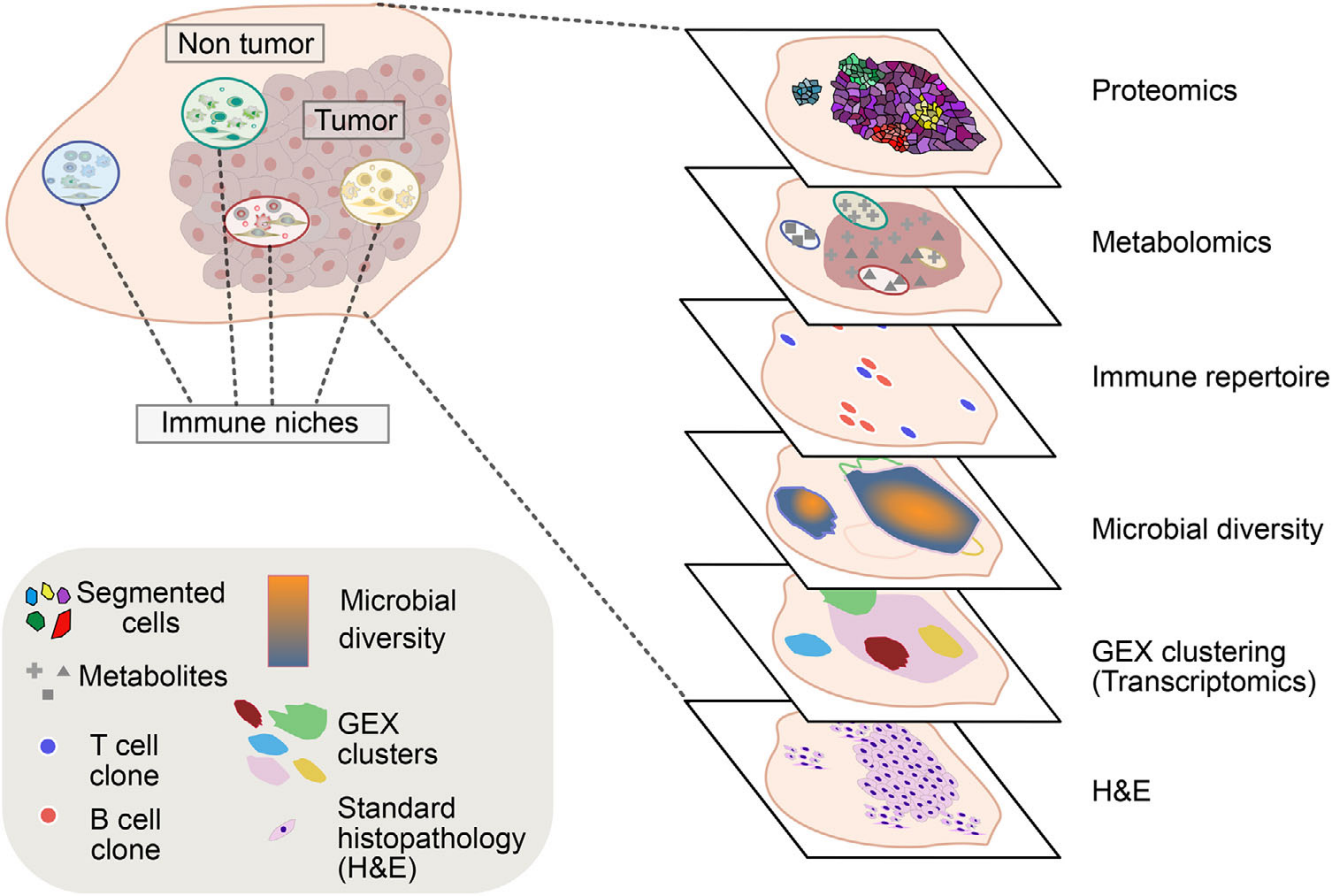
Illustration of various modalities used for immune niche profiling, shown from bottom to top: conventional H&E staining, gene expression clusters from spatial transcriptomics, microbial diversity from spatial metatranscriptomics, immune repertoire profiling, metabolites from spatial metabolomics, and cell segmentation output from spatial proteomics.

By combining sequencing‐based spatial transcriptomics with antigen receptor analysis, it is also possible to map the distribution and functional states of specific B‐ and T cell clones within their tissue niches (Figure 3) [12, 13, 14, 31]. For example, considering the emerging evidence of B and PC involvement in IBD, spatially resolving PC clones in the gastrointestinal tract of IBD patients could help identify putative pathogenic antibody‐expressing cells and uncover potential alterations within their local niche environment. Similarly, identifying distinct antigen receptors within GC during productive immune responses toward vaccines or in tissue lesions in autoimmunity could also help link specific B‐ or T cell clonal location to their antigen. In the future, it may be possible that knowing the location of a particular B and T cell clone could help resolve the antigen specificity, due to what is expressed in their niche, although it is not yet technically feasible. A limitation of the technology is also that it relies on gene expression, which facilitates the capture of more highly expressed B cell receptors by PCs, but can limit the capture of lower expressed B and T cell receptors. Combined, existing technologies, future improvements to capture sensitivity, for instance by in situ sequencing approaches [107], and applications of spatial antigen receptor analysis modalities will help further elucidate the interplay between clonal dynamics and the tissue‐based niche factors. More concretely, results from these types of analyses may create new avenues to boost tissue‐resident B and T cell clonal expansion by modulating important tissue niche factors or identifying key antigen receptor sequences based on tissue location that could be harnessed for therapy.

Beyond profiling the transcriptome, spatial multiomic approaches add complementary layers such as proteomics, metabolomics, epigenomics, genomics, and the microbiome, capturing putative critical molecular regulators of immune niches not accessible through host transcriptome analysis alone (Figure 3). Optimized tissue preparation protocols have also been developed to maximize capture area usage, e.g., placing the whole murine gastrointestinal tract in a single slide [108]. A recently developed framework, MicroCart, integrates transcriptomics, proteomics, glycomics, and bacterial detection from serial tissue sections to study host–microbiome interactions. It overlayed these data types using correlation network analysis to reveal cross‐modal features [109]. In parallel, new efforts aim to combine multiple modalities on the same tissue section, such as spatial transcriptomics with spatial metabolomics [110] or with spatial proteomics that can boost cellular identification for cell types with low transcriptional activity [111, 112]. Similarly, spatial meta‐transcriptomics enables the simultaneous detection of host, eukaryotic, and prokaryotic transcripts in tissues [113]. These types of analyses are particularly relevant in the context of the gastrointestinal tract, where the microbiome profoundly influences immune responses and vice versa, within distinct spatial environments [114, 115]. The tumor‐associated microbiome may also modulate the immune response and has been associated with treatment response and patient survival [116], although the relevance of microbial presence in nongastrointestinal tumors has been more controversial. A recently developed framework called “multiomic and ecological spatial analysis (MESA)” can be used to define spatial patterns across modalities by combining spatial and single‐cell data in a workflow inspired by ecological concepts [117]. By tying back to ecology, the field that inspired the use of niches in medical science can enable investigations of important immune niche features that cannot be inferred by the transcriptome alone. Combined, combined spatial meta‐ and multiomics offer a powerful way to perform multidimensional investigations of immune niches.

Lastly, spatial transcriptomics analysis of immune niches or findings derived from such analyses could be applied in the clinic [118]. For instance, key immune niches, rather than individual cell or molecular markers, may serve as biomarkers or guide precision medicine approaches. Despite the clinical success of cancer immunotherapy, lack of response, adverse events, and resistance limit its applicability [119, 120]. Identifying functionally relevant immune niches could help better understand the mechanism of response or resistance to therapy and predict therapeutic outcomes. Extending to other diseases, like IBD, where chronic microenvironments drive pathology, immune niches mapped in the diseased conditions could inform targeted interventions [121]. However, translation of complex niches to clinically implementable readouts can be challenging and not realistic due to cost, time, and complexity of the analysis. Distilling transcriptome‐wide information or large panels into a limited number of targets using machine learning or similar approaches could facilitate their application to patients. Similarly, recent advances that enable spatial transcriptomics analysis of FFPE samples are important for clinical translation since this type of tissue preservation is used more routinely in the clinic [118]. Currently, spatial transcriptomics methods also remain relatively expensive compared with other techniques commonly used in clinical investigations. However, broader adoption may drive down costs over time—mirroring the trajectory of sequencing technologies as they become integrated into clinical practice. Additionally, future advancements in spatial techniques can help amplify the clinical impact of immune niches, enabling more effective, personalized treatments.

## 3 Conclusion

Spatial transcriptomics has emerged as a critical tool for resolving the molecular and cellular architecture of the immune niches, uncovering how local environments influence immune behavior in both health and disease. Recent advances in spatial transcriptomics, along with increasingly sophisticated computational methods, have progressively refined our ability to identify, characterize, and interpret both established and previously unrecognized niches. Delineating immune niches using spatial transcriptomics, coupled with mechanistic studies and more precise conceptual definitions, has the potential to expand our understanding of immunological mechanisms in both health and disease, with the goal of accelerating precision medicine and finding new targeted therapies.

## Author Contributions

All authors wrote and edited the manuscript.

## Conflicts of Interest

Camilla Engblom was a scientific consultant for 10x Genomics, Inc., and is a patent holder on some of the technology described here. Eduardo J. Villablanca has been a paid consultant for Pfizer, Kancera AB, NanoString, Novome Biotechnologies, Ono Pharma, Mabylon, and Ferring. Eduardo J. Villablanca has received sponsor research support from Mabylon and F. Hoffmann‐La Roche AG and is the founder of PaperVids.

## Data Availability

No new datasets were generated for this review.
